# Infra-Low Frequency Neurofeedback: A Systematic Mixed Studies Review

**DOI:** 10.3389/fnhum.2022.920659

**Published:** 2022-07-12

**Authors:** Fabian Bazzana, Sarah Finzi, Giulia Di Fini, Fabio Veglia

**Affiliations:** Department of Psychology, University of Turin, Turin, Italy

**Keywords:** neurofeedback, infra-low frequency, ILF, slow brain activity, systematic review, PRISMA, MMAT

## Abstract

**Introduction:**

Neurofeedback training is increasingly applied as a therapeutic tool in a variety of disorders, with growing scientific and clinical interest in the last two decades. Different Neurofeedback approaches have been developed over time, so it is now important to be able to distinguish between them and investigate the effectiveness and efficiency characteristics of each specific protocol. In this study we intend to examine the effects of Neurofeedback based on slow brain activity, the so-called Infra-Low Frequency (ILF) training a recently developed methodology that seems promising for the regulation of the central nervous system.

**Aims:**

With this review we intend to summarize the currently existing literature on ILF-Neurofeedback, examine its quality and formulate indications about the clinical effectiveness of ILF-Neurofeedback.

**Methods:**

Literature search was first conducted according to PRISMA principles, described, and then assessed using the MMAT appraisal tool. 18 well-documented studies of ILF-Neurofeedback training in human subjects were picked up and analyzed. Reports include group interventions as well as single case studies.

**Results:**

Research data indicates good potential for ILF-Neurofeedback to influence brain activity and neurovegetative parameters. From the clinical profile, a salient common observation is a high level of individualization as a specific characteristic of ILF-Training: this feature seems to correlate with effectiveness of ILF-Neurofeedback, but also poses a challenge for researchers in terms of producing controlled and comparable findings; according to this point, some recommendation for future research on ILF-Neurofeedback are proposed. In conclusion, ILF-neurofeedback shows great potential for application for all those conditions in which the regulation of brain activity and neurophysiological processes are crucial. Further research will make it possible to complete the available data and to have a broader overview of its possible applications.

## Introduction

### Neurofeedback

**Figure 1:**
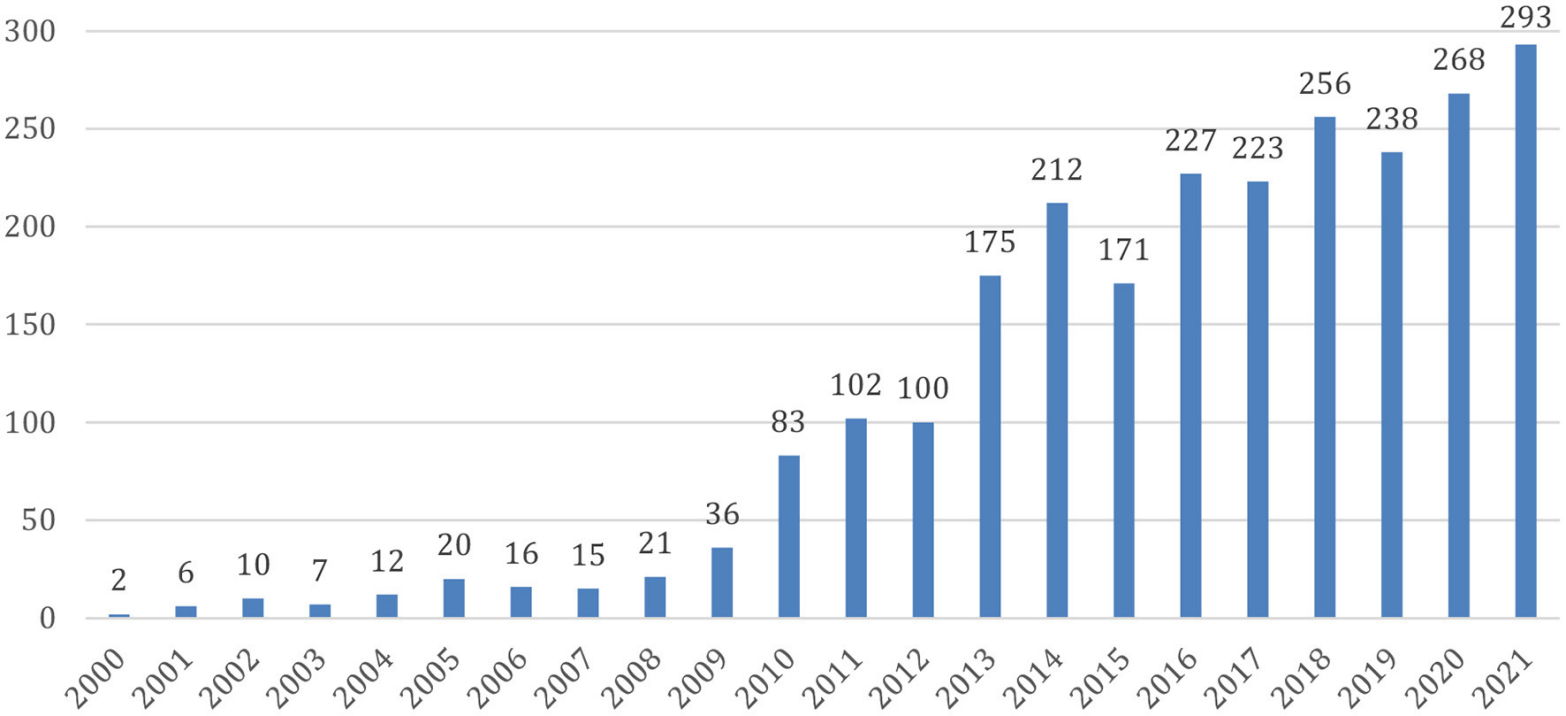
Published research articles about Neurofeedback. Source: Pubmed (https://pubmed.ncbi.nlm.nih.gov/); data access 15.01.2022.

Since Hans Berger discovered electrical brain activity in the 1920's, interest in brain waves has mushroomed, especially with regard to understanding the correlations between electrical activity of the brain, mental states and diseases. Neurofeedback is a kind of biofeedback in which brain waves are the targeted physiological parameter. Milestones in the development of the field include works of Kamiya (1969) and Sterman and Friar (1972) in the 60's and 70's. Based on an operant conditioning paradigm, Kamiya and Sterman both documented that the brain can alter its own activity through a system wherein brain waves are measured and a feedback signal is provided (acoustic, visual, or both). Sterman et al. (1978) also showed that enhancing a particular type of brain activity (the so-called sensorimotor rhythm, SMR) through Neurofeedback significantly reduced chemically induced seizures in cats. This was then replicated in monkeys. The first application to humans was published in a case report (Sterman and Friar, 1972); group studies followed, and a later review identified some 20 such studies, 13 with competent controls (Sterman, 2000). Since then, Neurofeedback has been utilized for a multitude of indications and symptoms (for a review, see Marzbani et al., 2016), and the number of publications on this topic has grown substantially over the last decade (Figure 1).

### Infra-Low Frequency Neurofeedback Training

While mainstream research on Neurofeedback was focused primarily on brain waves in the 8–25 Hz range (Alpha and Beta-band activity), Birbaumer et al. (1990) investigated slow potential shifts (*Slow Cortical Potentials, SCP*) that are related to motor and cognitive preparation. SCP Neurofeedback-Training was then applied to reduce cortical excitability. These findings introduced a modulation model of the brain that contributed to a more complex understanding of brain activity.

The discovery in 1996 of the existence of preferential training frequencies (outside of the obvious Alpha rhythm and the sleep spindle frequency) led to the finding of optimal response frequencies (ORF) across the broader EEG spectrum. The search for ORFs in each client eventually led to entry into the *Infra-Low Frequency* (ILF) regime, i.e., below 0.1 Hz (Othmer et al., 2013). The method became one of waveform-following, characterized by the absence of any discrete reinforcers. The realm of what are referred as Infra-slow Fluctuations (ISF) can also be trained by way of conventional operant conditioning. A convergence is becoming apparent between clinical findings based on ILF-Neurofeedback and neurological studies showing the association between slow brain activity fluctuations and a variety of neurophysiological processes, such as heart-rate variability, blood pressure and oxygenation and cortical excitability. These reports suggest a higher-order role of ILF-Rhythms in the self-organization and -regulation of the central nervous system (Grin-Yatsenko et al., 2020).

In parallel with the growth in clinical evidence, new hardware and software was developed to capture slow frequency oscillations and enable training below 0.1 Hz. This new training modality highlighted the need to rethink the mechanism of action that underlies the Neurofeedback: indeed, the basic operant conditioning conception was no longer suitable for explaining ILF-Neurofeedback training, given the absence of overt reinforcers. A more naturalistic model has been suggested, in which the brain acts to bring about closure between its expectation for the signal and the actual signal, in line with its normal self-regulation response (Othmer, 2015b; Othmer and Othmer, 2020). Clinical results and theoretical observations implicated ILF-Neurofeedback training in arousal regulation, thus engaging our intrinsic control networks, the salience network (SN) and the default mode network (DMN). Indirect validation of the conceptual assumptions of ILF-Neurofeedback comes from the concordance found between the empirically derived electrode placements, based on clinical effectiveness, and hubs of the DMN that are accessible at the cortical surface (Buckner et al., 2008; Othmer, 2015a). These are also the multi-modal association areas, which serve as input to the salience network. Thus the training engages two of the three networks identified by Menon (2011) for his Triple Network Model of Psychopathology. Clinical evidence, complemented with an appealing model, stimulated the present literature review on ILF Neurofeedback training.

## Purpose of This Review and Methods

As briefly mentioned above, research on slow neuronal and physiological patterns of activation point to a prominent role of these rhythms in regulating central nervous processes and large network patterns of activation. Clinical observations have highlighted the effectiveness of Neurofeedback that engages very slow oscillations in brain activity, therefore, an overview of the studies in this field and the data collected so far is of general interest.

### Aim of This Study

With this review, we intend first to collect all the available research findings in English relative to Infra-Low Frequency and Infra-Slow Fluctuations Neurofeedback, based on selected published clinical studies on healthy or clinical subjects. We believe this is the first systematic review to date that deals specifically with Infra-low Frequency Neurofeedback. Secondly, we wish to analyze the quality of the studies considered, using a method that allow us to compare disparate research designs. Finally, based on the parameters provided by the authors, we intend to consider the effectiveness of ILF-Neurofeedback as a clinical intervention tool. Possible directions for future research in this area will also be proposed based on the findings.

### Literature Search and Studies Selection

Literature search (in English, unlimited publication period) performed with PubMed, ResearchGate, PsycInfo, and PsycArticles, led to 36 pertinent articles. Key words selected for research were: “Infra-Low/Infra-Slow Frequency Neurofeedback”; “Neurofeedback *and* ILF/ISF”; “Neurofeedback *and* slow brain activity”. Literature research was conducted during January-February 2022. In addition, a literature list of 33 works was found on internet-site about ILF-Neurofeedback training, and in book bibliographies.

**Figure 2:**
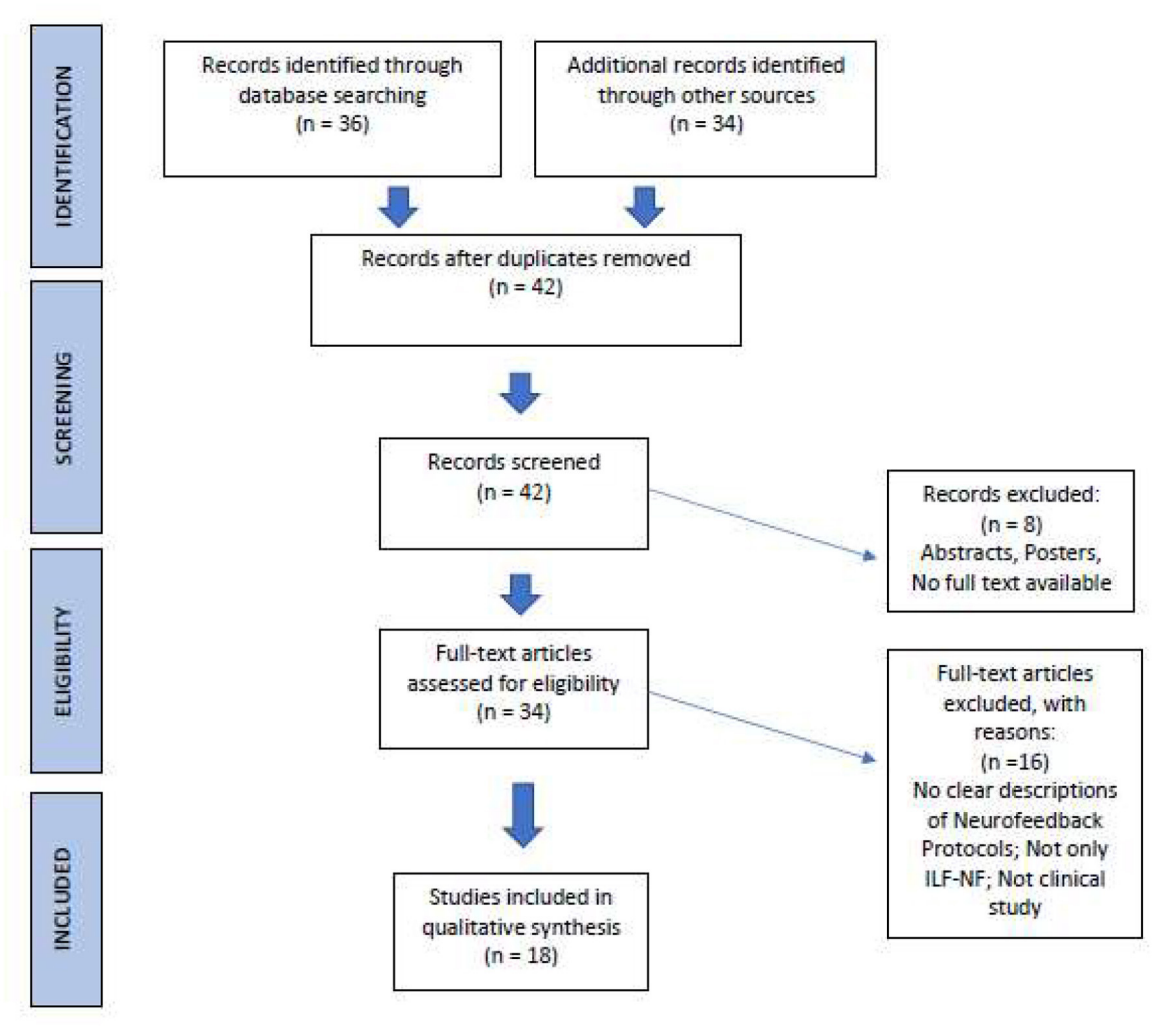
PRISMA process of literature selection (Page et al., 2021; http://prisma-statement.org/).

The publications we found underwent a selection process according to PRISMA statement (Page et al., 2021; Figure 2). After duplicates removal, criteria for eligibility were: (i) Clinical studies with clearly described ILF/ISF-Neurofeedback training as the sole intervention; and (ii) Use of objective parameters (neurobiological data, test, questionnaires, etc.) to determine the effectiveness of Neurofeedback training.

Group interventions as well as single case studies were included. Due to the small number of articles found, we have also included three well documented, university master or doctoral dissertations, accepted and published by the respective academic institutions (Nilsson and Nilsson, 2014; Ingvaldsen, 2019; Lamprecht, 2019). After comparing and selecting the papers, we came up with 18 studies. **Table 2** shows the selected studies and their main characteristics. One criterion that proved to be particularly discriminating concerned the precise description of the neurofeedback protocol used, since we had to ensure that the studies concerned ILF activity.

### Studies Assessment

The second step of our review involved the application of a tool to appreciate the quality of the selected studies. For this purpose, taking into account the variety of methodologies used in the selected studies, we used the Mixed Methods Appraisal Tool (MMAT, Hong et al., 2018). This procedure involves, after defining the type of study analyzed, the evaluation of specific research criteria by means of two general and five specific questions, to which the answers can be “yes,” “no” or “can't tell” (Table 1). Selected clinical studies underwent MMAT appraisal analysis through two independent examiners. After comparison and discussion, a score table was produced (**Table 3**).

**Table 1:** MMAT quality appraisal criteria description^a^.

**Category of study designs**	**Methodological quality criteria**
Screening questions (for all types)	S1. Are there clear research questions?
	S2. Do the collected data allow to address the research questions?
1. Qualitative	1.1. Is the qualitative approach appropriate to answer the research question?
	1.2. Are the qualitative data collection methods adequate to address the research question?
	1.3. Are the findings adequately derived from the data?
	1.4. Is the interpretation of results sufficiently substantiated by data?
	1.5. Is there coherence between qualitative data sources, collection, analysis and interpretation?
2. Quantitative randomized controlled trials	2.1. Is randomization appropriately performed?
	2.2. Are the groups comparable at baseline?
	2.3. Are there complete outcome data?
	2.4. Are outcome assessors blinded to the intervention provided?
	2.5 Did the participants adhere to the assigned intervention?
3. Quantitative non-randomized	3.1. Are the participants representative of the target population?
	3.2. Are measurements appropriate regarding both the outcome and intervention (or exposure)?
	3.3. Are there complete outcome data?
	3.4. Are the confounders accounted for in the design and analysis?
	3.5. During the study period, is the intervention administered (or exposure occurred) as intended?
4. Quantitative descriptive	4.1. Is the sampling strategy relevant to address the research question?
	4.2. Is the sample representative of the target population?
	4.3. Are the measurements appropriate?
	4.4. Is the risk of non-response bias low?
	4.5. Is the statistical analysis appropriate to answer the research question?
5. Mixed methods	5.1. Is there an adequate rationale for using a mixed methods design to address the research question?
	5.2. Are the different components of the study effectively integrated to answer the research question?
	5.3. Are the outputs of the integration of qualitative and quantitative components adequately interpreted?
	5.4. Are divergences and inconsistencies between quantitative and qualitative results adequately addressed?
	5.5. Do the different components of the study adhere to the quality criteria of each tradition of the methods involved?

a *Hong et al. (2018)*.

## Results

### Study Selection

The study selection process conducted according to PRISMA principles identified 18 papers suitable for our review, they are collected in Table 2.

**Table 2:** Selected articles summary by year of publication.

**N**.	**References**	**Topic**	**Sub-jects**	**Controls**	**Content**	**Sessions**	**Electrode positions**
14	Legarda et al. (2011)	Epilepsy, palsy	3	No	Three single cases of ILF-NF training of children with various neurological disorders.	21	T3-T4; C3-C4; T4-P4; T4-FP2
5	Chirita-Emandi and Puiu (2014)	Obesity	12	22	ILF-NF training in childhood obesity management.	20	T3-FP1
16	Nilsson and Nilsson (2014)	PTSD	12	9	Effects of ILF-NF training on PTSD-symptoms in traumatized refugees.	8–10	T4-P4; T3-T4
1	Altan et al. (2016)	ECG, EEG, GSR	40	No	Effects of one session of ILF-NF on Electroencephalogram (EEG), electrocardiogram (ECG) and galvanic skin resistance (GSR).	1	T3-T4
9	Grin-Yatsenko et al. (2018a)	Brain function-stress	8	No	Effects of ILF-NF on objective brain parameters by healthy adults.	20	T3-T4; T4-P4 T4-FP2; T3-FP1
11	Grin-Yatsenko et al. (2018b)	Depression	3	No	Three single cases of ILF-NF training of adults with depression.	20	T4-P4; T4-Fp2 T4-T3; T3-Fp1
15	Leong et al. (2018)	Eating disorders	11	12	Effects of ISF-NF compared to placebo on brain activity and food craving.	6	
12	Ingvaldsen (2019)	Fibromyalgya	13		Effects of ILF Neurofeedback on fibromyalgia (FM) affected patients as evaluated by qEEG and symptom scales.	10–15	T3-T4
13	Lamprecht (2019)	Concussion	7	9	Effects of ILF-NF training in concussion injury.	4	T3-T4; O1-O2
2	Balt et al. (2020)	Anxiety	20	9	Effects of ISF-Neurofeedback vs. SMR-NF on autonomic nervous system parameters in adults with anxiety.	10	T3-T4; T4-P4
6	Corominas-Roso et al. (2020)	Impulsivity	10	10	ILF-NF training in the treatment of impulsive behavior in long-term abstinent cocaine and heroin addicts.	40	T3-T4; T4-P4 T4-FP2; T3-FP1
7	Dobrushina et al. (2020)	Brain function	27	26	Resting-state fMRI parameters and connectivity patterns after a single session of ILF-NF or sham-NF.	1	T4-P4
8	Gerge (2020)	PTSD	1	no	ILF-NF Training in a Complex-PTSD client.	10	T4-P4; T4-FP2
10	Grin-Yatsenko et al. (2020)	Brain function - attention	9	8	Effects ILF-NF vs. HRV training by means of attention test and EEG oscillations.	20	T3-T4; T4-P4 T4-FP2; T3-FP1
3	Bekker et al. (2021)	Insomnia	20	20	Effects of ISF-NF on autonomic nervous system, cognitive and emotive parameters in adults with and without Insomnia.	10	T3-T4
4	Carlson and Webster Ross (2021)	Mild brain injury	4	No	ILF-NF effect on chronic headache, sleep and attention disorders in Veterans with Mild Traumatic Brain Injury.	20	T3-T4; T4-P4 T4-FP2; T3-FP1
17	Orakpo et al. (2021)	Pain	1	No	Single-case of ILF-NF in a client with pain and anxiety, sleep, depression and PTSD symptoms.	20	T3-T4; T4-FP2
18	Schneider et al. (2021)	ADHD	196	No	Effects of ILF-Neurofeedback sessions on attention, hyperactivity and impulsivity.	M = 38.5	T3-T4; T4-P4

The research articles found cover a total period of 10 years; it can also be noted that half of the studies were published within the last 2 years, reflecting the growing interest in ILF-Neurofeedback training.

### Narrative Description of Studies

Below we briefly summarize the main contents of the reviewed studies.

#### Infra-Low Neurofeedback and Brain Activity

A first group of articles (1, 7, 9, 10) has in common the investigation of the effects of ILF-Neurofeedback on brain activity in healthy subjects. Grin-Yatsenko et al. (2018a) found a significant increase of spectral power in the EEG 0.016–0.5 Hz frequency band after 20 sessions of ILF-Training in eight healthy subjects, compared to the pre-training EEG. They concluded that improvement of slow brain activity was linked to an improvement in the metabolic balance in brain tissue, and an increased efficiency of compensatory mechanisms in stress regulation systems.

In another research project, Grin-Yatsenko et al. (2020) selected 17 participants (9 randomly assigned in the research group and 8 controls) who had no diagnosable dysfunction, but reported some psychophysiological complaints (such as anxiety, mood swings, stress, fatigue, pain and sleep problems). After 20 Neurofeedback sessions in the ILF-EEG range, the experimental group displayed a significant increase of the power of brain EEG activity in the 0.01–0.5 Hz frequency band; same increase was not detected in the control group that received only HRV (Heart Rate Variability)-Biofeedback training. A greater health improvement was observed in the Neurofeedback group.

Dobrushina et al. (2020) examined fMRI patterns following a single ILF-Session in 27 healthy subjects, and found increased connectivity between key regions of the salience, language and visual networks. This suggested a functional integration of the large-scale brain networks involved in sensory processing. In the sham-Neurofeedback control group (26 participants), no such increase in brain connectivity was detected. It should be noted that the control group also showed changes in brain connectivity, indicating that the Sham-Neurofeedback cannot be assumed to be a completely neutral condition (see also the discussion in MMAT Quality Assessment).

Altan et al. (2016) observed an increased Beta activity and diminished alpha activity in the qEEG parameters during and after a single ILF-Neurofeedback session, highlighting the role of ILF-Training in restoring the neurovegetative balance. These studies suggest that objective changes occur in brain activity as a result of ILF-Neurofeedback training. As expected, an increase in the amplitude of infra-slow oscillations was reported. Other features, such as changes in connectivity patterns on large neuronal networks, or modifications of physiological parameters were also observed. Although not the primary objective of these studies, several participants also reported improvement in their wellbeing and psychophysiological balance. In light of these results, authors called for further controlled studies to gain more data, clarify the mechanism of action, further understand the influence of ILF-Training, and verify their long-term effects.

#### Infra-Low Neurofeedback and Symptom Treatment

The second group of studies is characterized by the application of ILF-Neurofeedback to various psychophysiological symptoms. As described above, classic Neurofeedback has been applied over the years as a therapy for a wide variety of disorders, e.g., applications in the field of ADHD and epilepsy. Given the promise of an effective arousal-regulating effect, ILF-Neurofeedback has also been the subject of trials for various types of disorders in recent years. The studies reviewed provide a good example of the state of the art in the clinical setting.

##### Depression

Depression is a mental disorder that negatively affects feelings, thinking and behavior. In many cases people affected by depression experience feelings of sadness, anxiety, loss of interest, and cognitive impairments such as memory loss or attention deficits. Depression also affects an individual's level of functioning at home as well as at work, and is a frequent cause of an inability to work (American Psychiatric Association, 2013). In 2017, major depressive episodes were estimated to be prevalent in 7.1% of adults in the USA (NIMH, 2017).

Grin-Yatsenko et al. (2018b) presented three case studies in which they evaluated the effects of infra-low neurofeedback on depression. One female and two male participants manifested depressive symptoms; none had consulted a doctor or taken antidepressive medication. Baseline investigation consisted of Depression Rating Scales: Montgomery-Åsberg Depression Rating Scale (MADRS), Hamilton Depression Rating Scale (HAMD), and Beck Depression Inventory (BDI). ILF-Neurofeedback was used with electrode positioning at T4-P4 and T4-Fp2 during the first sessions; subsequently electrodes were added at T4-T3 and T3-Fp1. After 20 Neurofeedback sessions, each 30–45 min long, all three patients exhibited an improvement of mood and self-organization skills, decreased anxiety, as well as increased emotional stability and resilience. In particular, the depression profile score of Cases A and B during the second testing changed, with all scales having improved by at least 90%; Case C showed an improvement of at least 70%. The depression profile score for all three participants did not indicate any depression and the improvements were stable for 1 year after beginning the ILF neurofeedback therapy. According to the authors, this study also showed that the training led to a change in brain activity, especially a decrease of theta activity over frontal and central areas in passive states (Grin-Yatsenko et al., 2018b).

##### Post-traumatic Stress Disorder

Post-traumatic stress disorder (PTSD) affects people who have experienced or assisted in a shocking, traumatic event where their physical or mental integrity is threatened. Symptoms may include flashbacks of the event, and disturbing thoughts, feelings and nightmares. A dysregulation of arousal is often associated with PTSD.

Nilsson and Nilsson (2014) conducted a pilot study with traumatized refugees who had been exposed to war and/or torture. The study was done in cooperation with the Red Cross Center for Victims of War and Torture in Malmö, where the Neurofeedback training was performed. Twenty-one individuals were divided into treatment-group (*n* = 12) or control-group (*n* = 9). Treatment consisted of 8-10 sessions of ILF-Neurofeedback, with initial electrode positions in T3-T4 or T4-P4, for 10–15 weeks. Five instruments were used (the PTSD Checklist: Civilian Version; the Hopkins Symptom Checklist−25; the Symptom Checklist: Subscale Somatization; the WHO-5—Wellbeing Index; and the Pittsburgh Sleep Quality Index) to measure differences in symptom severity. The results indicated a significant improvement on 4 of the 5 scales seen over time for the treatment-group compared to the controls. The authors reported some limitations of the research, in particular the non-homogenous control group.

Gerge (2020) describes the beneficial effects of training with 10 sessions of ILF-Neurofeedback for a patient with complex PTSD with a high degree of distress. In addition to the subjective improvement reported by the patient, improvements reported at several standardized scales are reported.

##### ADHD, Autism

Schneider et al. (2021) analyzed the results of ILF-Neurofeedback training used on 196 children (average age 12.1 years) with a diagnosis of ADHD, at 5 therapy centers in Germany. According to the Othmer ADHD treatment protocol (Othmer, 2015a), initial electrode positioning was at T3-T4 or T4-P4; training frequency was individually determined. After an average of 38.5 ILF-Neurofeedback sessions, the authors detected an improvement in attention, hyperactivity and impulsivity symptoms, and in performance on a continuous attention test (QIKtest, BeeMedic, Germany), which measures reaction time, as well as omission and commission errors. Due to the way the data were collected, no control group was included.

Legarda et al. (2011) evaluated neurofeedback training and its efficacy in modulating neurological activity by developmental disorders in three case studies. Only one (case B) was trained steady in the Infra-Low frequency range (down to 0.001 Hz); subject was a 6-year-old child born prematurely and suffered from cerebral palsy, autism, mental retardation, and symptomatic epilepsy managed by medication. After 21 ILF-Neurofeedback sessions, authors found an improvement in the autism profile by 63% and the onset of a normal sleep cycle.

##### Neurological and Psychophysiological Disorders

Several studies (4, 12, 13, 17) reported ILF-Training efficacy treating a wide range of neurophysiological symptoms and disorders, including migraine, fibromyalgia, multiple sclerosis, post-concussion syndrome and traumatic brain injury.

In a small pilot study, Carlson and Webster Ross (2021) treated four disabled male war veterans, 35–56 years, who reported headaches, insomnia, attention difficulties and general somatic and psychological symptoms due to traumatic brain injury during deployment; selection of participants was influenced by time constraints. Participants received 20 sessions three times weekly of ILF-Neurofeedback, with individually defined optimal frequency and positioning, starting at T3-T4 for stabilization and then adding T4-P4, T4-FP2, and T3-FP1. Result of the training was a significant symptom reduction in each of 12 the self-administered scales, like HIT-6 (Headache Impact Test), ISI (Insomnia Severity Index), PHQ-9 (Patient Health Questionnaire). Authors stated that no adverse effect could be observed and participants enjoyed the training.

Ingvaldsen (2019) explored the effects of ILF Neurofeedback on fibromyalgia-affected patients (FM). 13 females, previously diagnosed with FM, underwent qEEG examination and 10–15 ILF Neurofeedback sessions with electrode positioning at T3-T4. Five symptom scales with score measures were administered before and after NF training. The qEEG deviations normalized with ILF Neurofeedback, and fibromyalgia symptoms were reduced. The author suggested that ILF Neurofeedback affected symptom reduction by improving functional connectivity and information processing within and between pain networks.

Lamprecht (2019), examined the effect of ILF Neurofeedback on dynamic balance and complex gait with cognitive loading, compared to placebo, in young adults with post-concussion syndrome. 16 participants, students with diagnosis of post-concussion syndrome, underwent four sessions with either ILF-Neurofeedback (7) or sham-neurofeedback (9) with electrode positioning in T3-T4 and O1-O2. Dynamic and postural testing were performed prior to every session. As result, the Neurofeedback training group showed a significant positive effect on gait speed as well as on postural control, compared to the placebo group. Other measured variables did not differ between neurofeedback and placebo group.

Orakpo et al. (2021) reported a case of a 55-year-old woman, who presented with chronic pain related to post-concussive syndrome, sciatica and sequelae from a car accident, along with a history of depression, anxiety and PTSD related to the accident. The patient underwent 20 ILF-Neurofeedback sessions, with positioning at T3-T4 with frequency of 0.15 mHz and T4-P4 with frequency of 0.175 mHz. After the training, the women showed a significant decrease in pain intensity (from 6 to 4.5 points average) and anxiety, an improvement in daily activities and also a subtle improvement in sleep troubles and depression. The 1-year follow-up showed further decrease of the pain index.

##### Stress, Mood, and Neurovegetative Symptoms

Several studies have investigated the effects of neurofeedback by measuring physiological parameters and stress-related symptomatology (1, 2, 3, 9).

Altan et al. (2016) observed an increase in galvanic skin resistance and a decrease in heart rate in 40 healthy subjects, with a single session of ILF-Neurofeedback using T3-T4 electrode positioning.

Balt et al. (2020) showed significant changes in several neurophysiological parameters in a group of twenty adults with anxiety problems after 10 Infra-low frequency neurofeedback training sessions with electrode placement in T3-T4 or T4-P4. The same effects did not occur in the control group of nine subjects treated with sensorimotor rhythm (SMR) neurofeedback.

After 10 sessions of ILF-Neurofeedback with electrode placement in T3-T4, Bekker et al. (2021) observed significant changes in various neurovegetative parameters (skin temperature, heart rate, blood pressure), EEG activity, along with improved neurocognitive performance, in 40 subjects, divided in two groups with and without insomnia. They also reported a decrease in depression and in stress indices.

The above-cited work of Grin-Yatsenko et al. (2018a), showed positive effects of ILF-Training on 8 subjects with a wide list of symptoms (fatigue, depressed mood, symptoms of inner tension, mood swings, headache, sleep problems, diminished attention and poor working memory). Electrodes were placed at T4-P4, T4-T3 and, subsequently, T4-Fp2 and T3-Fp1. After 20 ILF-Neurofeedback sessions, all participants reported an improvement of their state: decrease of inner tension, reactivity to stressful factors, stability of mood, improved body and space awareness, increase of energy level and of cognitive performance.

##### Impulsivity Modulation

Corominas-Roso et al. recruited 20 inmates with a past cocaine and heroin addiction diagnosis (now abstinent) for a single-blind sham-controlled study to investigate the benefits of ILF-Neurofeedback on the modulation of impulsivity. Electrodes were placed at P4–T4, T3–T4, T4–Fp2, and T3–Fp1 for a total of 40 ILF-NF sessions. The optimal reward frequency was set between 0.01 and 0.02 mHz. After ILF-Neurofeedback training, clinical symptoms, such as depressive symptoms, anxiety, impulsivity and attention, had improved and the benefits were higher than in the control group. The authors suggested that Infra-low Neurofeedback was better than placebo in the modulation of impulsivity in the examined population (Corominas-Roso et al., 2020).

##### Eating Disorders

Leong et al. (2018) explored the effects of Infra-slow Neurofeedback on food craving in obese women with food addiction. The study was a randomized, double-blind, parallel trial. The research sample included 11 women and 10 control subjects, who, respectively, received Infra-Low Frequency Neurofeedback and sham-Neurofeedback for a total of six sessions. As hypothesis, the authors suggest that influencing posterior cingulate cortex (PCC) and the default mode network (DMM), could lead to benefits in eating management. After training, the authors found a significant increase in Infra-low activity in the PCC for the training group. They also found a benefit in two eating-disorder variables: a 39% decrease in intense desire to eat and a 36% decrease in the cognitive “anticipation of relief from negative states” produced by eating.

Chirita-Emandi and Puiu (2014) evaluated the ILF-neurofeedback training outcomes in 34 children with overweight and obesity, age 6–18 years. Twelve subjects were assigned in the intervention group and 22 in the control group. Assessment of children was done before the intervention and 3 and 6 months after the intervention. All participants received psychoeducational recommendations for weight management; intervention group also had 20 ILF-Neurofeedback sessions with electrode placement in T3-FP1. Quality of life improved similarly for both groups, whereas weight loss was higher in the control group than the intervention, which showed a quite high drop-out rate. Subjective outcomes reported by patients in the intervention group were less snacking, improved satiety, enhanced attention capacity, ameliorated hyperactivity, and better sleep patterns. As the author noted, this ILF-NF protocol with positioning in T3-FP1 differs from the original protocols guideline from Othmer (2015a), and this can be a reason for the quite weak results of the intervention group.

The analysis of the content of the selected studies shows a great variety of themes and methodologies applied. Overall, a great richness emerges both on the scientific and on the purely clinical level. In this sense, we can only confirm the interest reported by most of the authors for a deepening of clinical research with ILF/ISF-Neurofeedback.

### MMAT Quality Assessment

The MMAT assessment tool was applied to the selected studies by two independent observers. The respective judgments were then compared and in case of disagreement the study was jointly re-evaluated until consensus was reached. Table 3 shows the ratings of all the studies reviewed, grouped according to the type of methodology employed. For illustration purposes, an “overall MMAT score” for each study is also shown analogically, obtained by returning a full point for each “YES” response and an empty for each “NO” or “CAN'T TELL” response to the 5 specific questions. As the MMAT authors point out, such an index cannot solely be considered indicative of the general quality of the study, but rather can serve as a stimulus for discussion (Hong et al., 2018). Similarly, a percentage of positive answers for each MMAT question is shown at the bottom of the table.

**Table 3:** MMAT score table.

			**Quantitative randomized**					**Quantitative non-randomized**					**Quantitative descriptive**					**MMAT scoring**
**References**	**S1**	**S2**	**2.1**	**2.2**	**2.3**	**2.4**	**2.5**	**3.1**	**3.2**	**3.3**	**3.4**	**3.5**	**4.1**	**4.2**	**4.3**	**4.4**	**4.5**	
Balt et al. (2020)	Y	Y	C	N	N	N	Y											•◦◦◦◦
Chirita-Emandi and Puiu (2014)	Y	Y	C	N	Y	N	Y											••◦◦◦
Corominas-Roso et al. (2020)	Y	Y	C	Y	Y	N	Y											••◦◦◦
Dobrushina et al. (2020)	Y	Y	C	Y	Y	N	Y											•••◦◦
Grin-Yatsenko et al. (2020)	Y	Y	C	C	Y	N	Y											••◦◦◦
Lamprecht (2019)	Y	Y	C	C	Y	Y	Y											•••◦◦
Leong et al. (2018)	Y	Y	Y	Y	Y	Y	Y											•••••
Bekker et al. (2021)	Y	Y						N	N	Y	N	Y						••◦◦◦
Nilsson and Nilsson (2014)	Y	Y						C	N	Y	C	N						•◦◦◦◦
Altan et al. (2016)	Y	Y											N	N	Y	Y	Y	•••◦◦
Carlson and Webster Ross (2021)	Y	Y											N	N	Y	N	N	•◦◦◦◦
Gerge (2020)	Y	Y											N	N	Y	Y	N	••◦◦◦
Grin-Yatsenko et al. (2018a)	Y	Y											C	N	Y	Y	Y	•••◦◦
Grin-Yatsenko et al. (2018b)	Y	Y											N	C	Y	Y	Y	•••◦◦
Legarda et al. (2011)	Y	Y											C	Y	C	N	N	•◦◦◦◦
Ingvaldsen (2019)	Y	Y											N	N	Y	Y	Y	•••◦◦
Orakpo et al. (2021)	Y	Y											C	Y	C	N	N	•◦◦◦◦
Schneider et al. (2021)	Y	Y											C	Y	Y	Y	Y	••••◦
Positive response percentage			14	43	86	26	100	0	0	100	0	50	0	33	78	67	56	

The MMAT evaluation procedure led us to trace the studies analyzed back to three research methodologies (quantitative randomized, quantitative non-randomized and quantitative descriptive) and to highlight some general strengths and weaknesses with regard to the research conducted so far on ILF-Neurofeedback.

During the first stage of literature search, we encountered a good number of *single case experiments*. Even considering the limitations that this type of research entails, for the scope investigated they can represent an important source of inspiration, especially for clinical trials. Unfortunately, in many cases the lack of precise information (characteristics of the participant, methodology used, etc.) and standardized data, lead to exclusion from this review. In order for this important type of clinical research to reach its full potential, it is therefore imperative that it is applied according to the correct methodology (Lobo et al., 2017). The retained single-cases studies (4, 8, 11, 14, 17) give a good overview of the possible applications of ILF-Neurofeedback in the clinical setting, showing positive effects, mainly measured with subjective and objective instruments, in the face of severe symptoms.

In *quantitative randomized* study designs (2, 5, 6, 7, 10, 13, 15) we have a solid body of research that generally shows good clinical effectiveness of ILF-Neurofeedback compared to control groups. With respect to the methodology examined, a common observation was the lack of a clear description of the randomization method utilized, perhaps considered to be of secondary importance by the authors; only in one case (Leong et al., 2018) is the procedure precisely outlined. Another aspect that emerged when examining the *quantitative* (randomized, non-randomized, and descriptive) studies in detail, concerns the objective difficulty of implementing a classic double-blind research design, especially when there is the goal to optimize the clinical effects of Neurofeedback. Generally speaking, in studies involving a therapeutic instrument applied by a practitioner, it is difficult to carry out a true double-blind protocol. In addition to this, due to the particular methodology of ILF-Neurofeedback, electrode positions and training frequencies are adjusted on the basis of feedback from the participant, leading to individualized treatments. Other control methods, like waiting lists or placebo groups using sham-Neurofeedback, as was the case for some of the studies analyzed, are ethically dubious and only feasible in studies with a very limited number of sessions (like Leong et al., 2018; Lamprecht, 2019); this, however, limits the possibility of verifying the therapeutic effects on symptomatic samples, where, as a rule, more sessions are required to obtain a certain clinical efficacy. Furthermore, the effects of prolonged use of a “sham” type of ILF-Neurofeedback should be verified to avoid possible iatrogenic effects on participants. For these reasons, an *active concurrent control design* (as in Balt et al., 2020) should be considered whenever possible, as proposed by the Declaration of Helsinki (World Medical Association, 2018; Nair, 2019).

With regard to statistical analysis reported in the studies, on the one hand we can often observe very sophisticated mathematical tools for extraction and combination of data, while for their interpretation in terms of significance (for repeated measures or comparison between measures) authors fall back on *p*-value. In order to be able to interpret the data more reliably in quantitative studies, it would be advisable to consider a *power-analysis* to estimate the necessary sample size and to check, where possible, the *effect size* of the results obtained (McGough and Faraone, 2009).

Another important aspect concerns sampling strategy and representativeness of the sample chosen in *descriptive studies* (MMAT 4.1 and 4.2), especially when the research question concerns the therapeutic efficacy of Neurofeedback on a specific clinical population. Although in most studies the diagnostic process is adequate, participants often come spontaneously to the research, without selection, or belong to groups of people already under treatment by the study authors. This makes it difficult to assess whether the research participants can actually be considered representative of the clinical reference population. Of course, this is a general issue in all clinical studies. On the other hand, the studies analyzed present for the most part a comprehensive description of the methodologies used and objective and reliable measures of the variables investigated, allowing for an in-depth discussion of the data collected and a good replicability of the research designs.

In addition, the low number of drop-outs reported by the authors should be noted, even when the research design included a high number of Neurofeedback sessions (20 or more).

## Discussion

### State-of-the-Art Research on ILF-Neurofeedback

In this literature review of Infra-Low Frequency Neurofeedback, 18 studies were selected from a larger sample of research articles, according to PRISMA principles. An initial selection of the literature concerned the possibility of ascertaining the effective use of slow brain activity as the training parameter for Neurofeedback. Inaccurate descriptions of the Neurofeedback method used, only partial indications of the data collected or concomitance with other therapeutic interventions led to exclusion. The final group of papers selected for this review, compared to the total number of publications on Neurofeedback in the last 10 years (over 2,000 from Pubmed searches alone), is a rather small sample even considering the relatively new development of ILF-Neurofeedback. However, the overall quality of the selected studies is to be considered good, in light of the application of the MMAT assessment tool. Authors show an important effort to produce solid methodologies on which to base data collection and answer research hypotheses. Some general limitations, as indicated in point 3.3, are related to the difficulty of producing proper controlled studies and ensuring the representativeness of the samples investigated. Despite these challenges, the findings resulting from the collected data converges toward a favorable clinical response to the application of ILF-Neurofeedback on brain activity, and many authors suggest that further research in this area is desirable.

### ILF-Neurofeedback, Specificity and Clinical Results

Following up on the objective of verifying the effectiveness of ILF-Neurofeedback, we have selected an initial group of research studies that focused on verifying its ability to modify slow brain activity. This has often been correlated with the modulation of several neurophysiological processes and with the level of sensitivity of large neuronal networks, especially the default mode network, as described above. The data collected from the trials on healthy subjects indicate that even with just one session of ILF-Neurofeedback, changes were observed in the basal brain activity and connectivity of the brain. Several parameters of neurovegetative activation (temperature, blood pressure, skin conductance, etc.) have also been shown to be significantly influenced by ILF-training (Section Infra-Low Neurofeedback and Brain Activity).

A group of studies concerned the application of ILF-Neurofeedback to clinical symptomatology. Positive effects have been highlighted in the literature reviewed for a wide range of symptoms and in general, a favorable effect on wellbeing has been reported (Section Infra-Low Neurofeedback and Symptom Treatment). Although more studies are desirable to confirm the observations, this wide application and versatility of ILF-Neurofeedback may suggest a general, non-specific mechanism of action toward a stress reduction effect and better brain modulation, according to the regulatory explication model proposed by Othmer and Othmer (2020). Conversely, if these results are further confirmed, the hypothesis of a common factor in a broad spectrum of the psychopathological symptomatology may well be supported.

What clearly emerges from many of the studies analyzed, and what distinguishes ILF-Neurofeedback from other methods, is the high degree of individualization of training protocols. The optimal training frequency is sought in most cases, followed by micro-adjustments during sessions and from session to session, based on client response. In addition, placement of electrodes rarely reduces to a single one; more typically 2–4 different positions are utilized in a varying way for each participant to maximize therapeutic effects. As reported, these features are intrinsic to ILF-Neurofeedback and its governing principles, thus differing significantly from classic EEG-Neurofeedback, which relies on “good” and “bad” frequencies, or Z-score Neurofeedback, targeting a “normalization” of brain activity (Haus et al., 2016).

The high level of individualization of the training, and the prominent role of the client involvement in the therapeutic process, may be reasons for the good therapeutic effectiveness reported in the studies examined, indicating that ILF-Neurofeedback is a neurophysiological tool whose effectiveness is correlated with—perhaps even contingent on—the quality of the client-therapist interaction. Implicitly, the neurofeedback practitioner must have very good technical *and* clinical expertise, correctly interpreting training effects and client reports. As a virtuous “side effect” of ILF-training procedure, increasing client awareness about their symptomatic and positive mind/body states may well aid the journey to mastery.

## Conclusions and Indications for Research

Referring to the objectives of this review, we can conclude that studies on ILF-Neurofeedback training are still relatively few at the moment, but of good quality. With regard to its efficacy, ILF-Neurofeedback has shown promising results for a wide variety of symptoms, promoting better regulation of brain activity and of the neurovegetative system.

Insofar as therapeutic effectiveness is correlated with individualization of ILF-Neurofeedback protocols, a challenge is posed to standard research designs. Researchers are confronted with a complex decision-making process, having to find a delicate equilibrium between maximizing therapeutic effectiveness (therefore individualizing protocols) and reducing variability to ensure validity and replicability of the studies. Considering the young age of ILF-Neurofeedback Training and the promising results rising from the reviewed literature, there is a scientific and clinical interest to motivate further controlled studies. The following are some indications for future research in this domain, mainly provided by the authors themselves or extrapolated from the analysis conducted in this review: (a) in group trials, where possible it is suitable to consider the representativeness of the sample for the reference population and the sample size in regard to the expected effects (power analysis); (b) in clinical studies, psychosocial data, along with diagnosis and symptomatology (and their variation) of participants should be clearly defined with objective and international validated instruments; (c) where possible, a control group with a competing, already scientifically validated treatment is desirable; (d) ILF-Neurofeedback protocols should be defined as clearly as possible (individualization can be made more replicable through an explicitly defined algorithm), (e) where therapeutic effects are investigated, a sufficient number of sessions is provided (at least 20 sessions); (f) a statistical measure of the magnitude of the effects found is provided (effect size); (g) objective measures of brain activity and its variation are provided, especially those targeted by the Neurofeedback intervention; (h) by single cases studies, the adoption of a standard like CARE-Guidelines (Gagnier et al., 2013) or SCRIBE-Guidelines (Tate et al., 2016) can be worthwhile.

These directions largely agree with the recent attempt to formulate general guidelines for research in Neurofeedback clinical studies, the CRED-nf checklist, formulated by Ros et al. (2020).

## Limits of This Review

This review was written with the purpose of collecting and summarizing available research work on ILF-Neurofeedback, a relatively novel procedure with implications for theoretical models of neuroregulation. The number of documented research studies is still quite small, and varying in study design, so any conclusion about ILF-Neurofeedback and its effectiveness would be premature.

Due to the limited time available, it was not possible to contact all the authors whose works presented gaps or inconsistencies with the selection criteria adopted for this review and verify them. We therefore apologize to all authors whose work was excluded from this study.

## Data Availability Statement

The original contributions presented in the study are included in the article/supplementary material, further inquiries can be directed to the corresponding author.

## Author Contributions

FB is responsible for research, writing, and editing. SF and GD contributed to review and editing. FV contributed to main supervision. All authors contributed to the article and approved the submitted version.

## Conflict of Interest

The authors declare that the research was conducted in the absence of any commercial or financial relationships that could be construed as a potential conflict of interest.

## Publisher's Note

All claims expressed in this article are solely those of the authors and do not necessarily represent those of their affiliated organizations, or those of the publisher, the editors and the reviewers. Any product that may be evaluated in this article, or claim that may be made by its manufacturer, is not guaranteed or endorsed by the publisher.

## References

[B1] Altan S. Berberoglu B. Canan S. Dane S. (2016). Effects of neurofeedback therapy in healthy young subjects. Clin. Invest. Med. 39, 27496. 10.25011/cim.v39i6.27496 27917787

[B2] American Psychiatric Association. (2013). Diagnostic and Statistical Manual of Mental Disorders, 5th Edn. American Psychiatric Association. 10.1176/appi.books.9780890425596

[B3] Balt K. Du Toit P. Smith M. van Rensburg C. J. (2020). The effect of infraslow frequency neurofeedback on autonomic nervous system function in adults with anxiety and related diseases. NeuroRegulation 7, 64–74. 10.15540/nr.7.2.64

[B4] Bekker M. Balt K. Bipath P. Jordaan J. du Toit P. (2021). The effect of infra-slow fluctuation neurofeedback training on a cohort of insomnia participants. NeuroRegulation. 8, 137–148. 10.15540/nr.8.3.137

[B5] Birbaumer N. Elbert T. Canavan A. Rockstroh B. (1990). Slow potentials of the cerebral cortex and behavior. Physiol. Rev. 70, 1–41. 10.1152/physrev.1990.70.1.1 2404287

[B6] Buckner R.L. Andrews-Hanna J.R. Schacter D.L. (2008). The brain's default network: anatomy, function, and relevance to disease. Ann. N. Y. Acad. Sci. 1124, 1–38 10.1196/annals.1440.011 18400922

[B7] Carlson J. Webster Ross G. (2021). Neurofeedback impact on chronic headache, sleep and attention disorders experienced by veterans with mild traumatic brain injury: a pilot study. Biofeedback 49, 1. 10.5298/1081-5937-49.01.01

[B8] Chirita-Emandi A. Puiu M. (2014). Outcomes of neurofeedback training in childhood obesity management: a pilot study. J. Alternat. Complement. Med. 20, 11. 10.1089/acm.2014.0040 25188371

[B9] Corominas-Roso M. Ibern I. Capdevila M. Ramon R. Roncero C. Ramos-Quiroga J. A. (2020). Benefits of EEG-neurofeedback on the modulation of impulsivity in a sample of cocaine and heroin long-term abstinent inmates: a pilot study. Int. J. Offender Ther. Compar. Criminol. 64, 1275–1298. 10.1177/0306624X20904704 32090660

[B10] Dobrushina O. Vlasova R.M. Rumshiskaya A.D. Litvinova L.D. Mershina E.A. Sinitsyn V.E. . (2020). Modulation of intrinsic brain connectivity by implicit electroencephalographic neurofeedback. Front. Hum. Neurosci. 14, 192. 10.3389/fnhum.2020.00192 32655386 PMC7324903

[B11] Gagnier J.J. Kienle G. Altman D.G. Moher D. Sox H. Riley D. (2013). The CARE guidelines: consensus-based clinical case reporting guideline. Glob. Adv. Health Med. 2, 38–43. 10.7453/gahmj.2013.008 24416692 PMC3833570

[B12] Gerge A. (2020). A Multifaceted case-vignette integrating neurofeedback and EMDR in the treatment of complex PTSD. Eur. J. Trauma Dissoc. 4, 100157. 10.1016/j.ejtd.2020.100157

[B13] Grin-Yatsenko V. Kara O. Evdokimov S. Gregory M. Othmer S. Kropotov J. (2020). Infra-low frequency neurofeedback modulates infra-slow oscillations of brain potentials: a controlled study. J. Biomed. Eng. Res. 4, 1–11. 10.1016/j.clinph.2019.12.091

[B14] Grin-Yatsenko V. A. Othmer S. Ponomarev V. A. Evdokimov S. A. Konoplev Y. Y. Kropotov J. D. (2018a). Infra-low frequency neurofeedback in depression: three case studies. NeuroRegulation 5, 30–42. 10.15540/nr.5.1.30

[B15] Grin-Yatsenko V. A. Ponomarev V. A. Kara O. Wandernoth B. Gregory M. Ilyukhina V. A. Kropotov J. (2018b). Effect of Infra-Low Frequency Neurofeedback on Infra-Slow EEG Fluctuations. IntechOpen. 10.5772/intechopen.77154

[B16] Haus K.-M. Held C. Kowalski A. Krombholz A. Nowak M. Schneider E. . (2016). Praxisbuch Biofeedback und Neurofeedback. Heidelberg: Springer.

[B17] Hong Q.N. Pluye P. Fàbregues S. Bartlett G. Boardman F. Cargo M. . (2018). Mixed Methods Appraisal Tool (MMAT), Version 2018. Available online at: http://mixedmethodsappraisaltoolpublic.pbworks.com/ (accessed May 1, 2022).

[B18] Ingvaldsen S. H. (2019). QEEG and Infra-Low Frequency Neurofeedback Training in Fibromyalgia: A Pilot Study. (dissertation/master's thesis). Trondheim (Norway). Norwegian University of Science and Technology. Available online at: https://ntnuopen.ntnu.no/ntnu-xmlui/handle/11250/2602751 (accessed January 10, 2022).

[B19] Kamiya J. (1969). Operant Control of the EEG Alpha Rhythm and Some of Its Reported Effects on Consciousness. Altered States of Consciousness. New York: Wiley, 1069.

[B20] Lamprecht C. E. (2019). The Effect of Neurofeedback in Post-concussion Syndrome. (dissertation/master's thesis). Stellenbosch (South Africa). Stellenbosch University. Available online at: https://scholar.sun.ac.za/handle/10019.1/106114 (accessed January 10, 2022).

[B21] Legarda S. B. McMahon D. Othmer S. S. Othmer S. S. (2011). Clinical neurofeedback: case studies, proposed mechanism, and implications for pediatric neurology practice. J. Child Neurol. 26, 1045–1051. 10.1177/0883073811405052 21576401

[B22] Leong S. L. Vanneste S. Lim J. Smith M. Manning P. De Ridder D. (2018). A randomised, double-blind, placebo-controlled parallel trial of closed-loop infraslow brain training in food addiction. Sci. Rep. 8, 1–9. 10.1038/s41598-018-30181-7 30076365 PMC6076277

[B23] Lobo M. A. Moeyaert M. Baraldi Cunha A. Babik I. (2017). Single-case design, analysis, and quality assessment for intervention research. J. Neurol. Phys. Ther. 41, 187–197. 10.1097/NPT.0000000000000187 28628553 PMC5492992

[B24] Marzbani H. Marateb H. R. Mansourian M. (2016). Neurofeedback: a comprehensive review on system design, methodology and clinical applications. Basic Clin. Neurosci. 7, 143–158. 10.15412/J.BCN.03070208 27303609 PMC4892319

[B25] McGough J. J. Faraone S. V. (2009). Estimating the size of treatment effects: moving beyond p values. Psychiatry 6, 21–29. 20011465 PMC2791668

[B26] Menon V. (2011). Large-scale brain networks and psychopathology: a unifying triple network model. Trends Cogn. Sci. 15, 483–506. 10.1016/j.tics.2011.08.003 21908230

[B27] Nair B. (2019). Clinical trial designs. Indian Dermatol. Online J. 10, 193–201. 10.4103/idoj.IDOJ_475_18 30984604 PMC6434767

[B28] Nilsson R. M. Nilsson V. (2014). Neurofeedback Treatment for Traumatized Refugees-A Pilot Study. (dissertation/master's thesis). Lund (Sweden). Lund University. Available online at: http://lup.lub.lu.se/student-papers/record/4459760 (accessed January 10, 2022).

[B29] NIMH (2017). Data from 2013 National Survey on Drug Use and Health. National Institute of Mental Health. Available online at: www.nimh.nih.gov/health/statistics/prevalence/major-depression-among-adults.shtml (accessed February 15, 2022).

[B30] Orakpo N. Vieux U. Castro-Nuñez C. (2021). Case report: virtual reality neurofeedback therapy as a novel modality for sustained analgesia in centralized pain syndromes. Front. Psychiatry 12, 660105. 10.3389/fpsyt.2021.660105 33959057 PMC8093562

[B31] Othmer S. (2015a). Protocol Guide for Neurofeedback Clinicians. Woodland Hills, CA: EEG Info Inc.

[B32] Othmer S. (2015b). History of neurofeedback, in Restoring the Brain, ed Kirk H. W. (Boca Raton, FL: CRC Press; Taylor and Francis Group). 10.1201/b18671-4

[B33] Othmer S. Othmer S. (2020). Toward a Theory of Infra-Low Frequency Neurofeedback. Augmented version of Chapter 3 in Restoring the Brain, 2nd Edn. Ed Kirk H. (Taylor and Francis). Available online at: http://www.eeginfo.com/research/pdfs/Toward-a-Theory-of-ILF-Neurofeedback.pdf (accessed March 12, 2022).

[B34] Othmer S. Othmer S. F. Kaiser D. A. Putman J. (2013). Endogenous neuromodulation at infralow frequencies. Semin. Pediatr. Neurol. 20, 246–257. 10.1016/j.spen.2013.10.006 24365573

[B35] Page M.J. McKenzie J.E. Bossuyt P.M. Boutron I. Hoffmann T.C. Mulrow . (2021). The PRISMA 2020 statement: an updated guideline for reporting systematic reviews. Int. J. Surg. 88, 1. 10.1016/j.ijsu.2021.105906 33789826

[B36] Ros T. Enriquez-Geppert S. Zotev V. Young K.D. Wood G. Whitfield-Gabrieli S. (2020). Consensus on the reporting and experimental design of clinical and cognitive-behavioural neurofeedback studies (CRED-nf checklist). Brain 143, 1674–1685. 10.1093/brain/awaa009 32176800 PMC7296848

[B37] Schneider H. Riederle J. Seuss S. (2021). Therapeutic effect of infra-low-frequency neurofeedback training on children and adolescents with ADHD. Intech 2021, 13. 10.5772/intechopen.97938

[B38] Sterman M. Friar L. (1972). Suppression of seizures in an epileptic following sensorimotor EEG feedback training. Electroencephalogr. Clin. Neurophysiol. 33, 89–95. 10.1016/0013-4694(72)90028-4 4113278

[B39] Sterman M.B. (2000). Basic concepts and clinical findings in the treatment of seizure disorders with EEG operant conditioning. Clin. Electroenceph. 31, 45–55. 10.1177/155005940003100111 10638352

[B40] Sterman M.B. Goodman S.J. Kovalesky R.A. (1978). Effects of sensorimotor EEG feedback training on seizure susceptibility in the rhesus monkey. Exp. Neurol. 62, 735–747. 10.1016/0014-4886(78)90281-9 108125

[B41] Tate R. L. Perdices M. Rosenkoetter U. Shadish W. Vohra S. Barlow D. H. Wilson B. (2016). The single-case reporting guideline in behavioural interventions (SCRIBE) 2016 statement. Arch. Sci. Psychol. 4, 1–9. 10.1037/arc0000026 27268573

[B42] World Medical Association (2018). Declaration of Helsinki: Ethical Principles for Medical Research Involving Human Subjects. Available online at: https://www.wma.net/policies-post/wma-declaration-of-helsinki-ethical-principles-for-medical-research-involving-human-subjects/ (accessed April 9, 2022).

